# A Case Report of Sepsis-Induced Dilated Cardiomyopathy Secondary to Human Metapneumovirus Infection

**DOI:** 10.7759/cureus.57951

**Published:** 2024-04-10

**Authors:** Toyin Ingram, Moses O Evbuomwan, Amudhan Jyothidasan

**Affiliations:** 1 Internal Medicine, Cape Fear Valley Health, Fayetteville, USA; 2 Radiation Oncology, University of Iowa, Iowa, USA; 3 Cardiology, Cape Fear Valley Medical Center, Fayetteville, USA

**Keywords:** dilated cardiomyopathy, sepsis-induced cardiomyopathy, viral endocarditis, myocarditis, human metapneumovirus infection, severe sepsis, sepsis

## Abstract

Sepsis is a medical emergency that describes the body's systemic immunological response to an infectious process that can lead to end-stage organ dysfunction and death. Sepsis-induced cardiomyopathy (SICM) is an increasingly recognized form of transient cardiac dysfunction characterized by left ventricular dilation, depressed ejection fraction, and recovery in 10 days without cardiac-related medical intervention. Injury to the myocardium by inflammatory cytokines has been proposed as one of the main causative mechanisms. Human metapneumovirus (hMPV) is a paramyxovirus and a common cause of respiratory tract infection that has been reported to modulate chemical mediators that produce inflammatory cytokines. Extra-pulmonary cardiac complications of hMPV have been reported; but literature on SICM associated with hMPV are very rare. We describe a case of a 43-year-old male with no known cardiac history diagnosed with SICM associated with hMPV. His sepsis was managed in the intensive care unit, and his heart ejection fraction improved within 10 days without the initiation of guideline-directed medical therapy.

## Introduction

Sepsis-induced cardiomyopathy (SICM) is a reversible myocardial dysfunction characterized by left ventricular dilation, depressed ejection fraction, and recovery in 7-10 days. There are two proposed causative mechanisms to explain sepsis-induced cardiac dysfunction which include (1) myocardial ischemia because of inadequate coronary blood flow and (2) chemical mediators producing inflammatory cytokines that cause injuries to the myocardium [1].

Respiratory viruses are known to modulate cytokine responses; but compared to respiratory syncytial virus and influenza, human metapneumovirus (hMPV) is a less effective inducer of different cytokines [2]. hMPV initially discovered in 2001 in the Netherlands is found globally and more common among the pediatric population with respiratory disease [2,3]. It has also been reported as causing respiratory disease in adults [2,4]. hMPV is spread predominately by respiratory droplets and individuals present with signs and symptoms of an upper and/or lower respiratory tract infection, with the latter being more common [2]. It has also been associated with extra-pulmonary manifestations including encephalitis, focal seizures, status epilepticus [5], and viral myocarditis [4].

Weinreich et al. reported the first case of hMPV causing viral myocarditis in 2015 [6], Choi et al. reported the second case in 2016 [7], and Bhatia et al. reported another case in 2023 [4]. Makhlouf et al. reported the first case of acute myocarditis caused by metapneumovirus in an immunocompromised 14-year-old girl [8]. A fatal triad of acute disseminated encephalomyelitis with seizures and myocarditis was reported in a 4-year-old girl infected with hMPV and picornavirus [5]. Also, a rare case of acute respiratory distress syndrome and myocarditis associated with hMPV in a 2.5-month-old boy was reported by Yakut et al. [9] in 2020. Whether hMPV has an affinity for myocardium or if patients with prior underlying cardiovascular disease are more susceptible to hMPV remains to be clarified. SICM associated with hMPV has not been reported. Here, we report a case of a 43-year-old male with no known cardiac history diagnosed with SICM secondary to hMPV infection.

## Case presentation

A 43-year-old male with a past medical history of gastric bypass (2021), chronic multivitamin infusion therapy, and no known cardiac issues presented to the emergency room (ER) with altered mental status. Per his spouse who provided a history of present illness, he had general malaise, chills, and diarrhea for two to three days, followed by new-onset cough, shortness of breath, and acute confusion before his ER arrival. No fever or rigor was reported. He works as a schoolteacher and has no recent travel and no exposure to bats, dead birds, rats, or rat droppings. He has not been in a crawl space, attic, or hot tub or encountered water-cooled AC units. No exposure to mold and substance and alcohol use were reported.

In the ER, he was noted to be hypotensive with a blood pressure of 80/54, a temperature of 103.3°F, a heart rate of 135 beats per minute, and a respiratory rate of 20 breaths per minute. He was also noted to be hypoglycemic with a blood glucose of 69. Physical examination revealed altered mental status and crackles on lung auscultation. Examination for JVD elevation, heart murmur, and peripheral lower extremities edema were negative. He was observed to be using accessory muscles for respiration and was unable to clear oral secretions. The patient was intubated for airway protection and respiratory support due to acute-onset metabolic encephalopathy.

**Figure 1:**
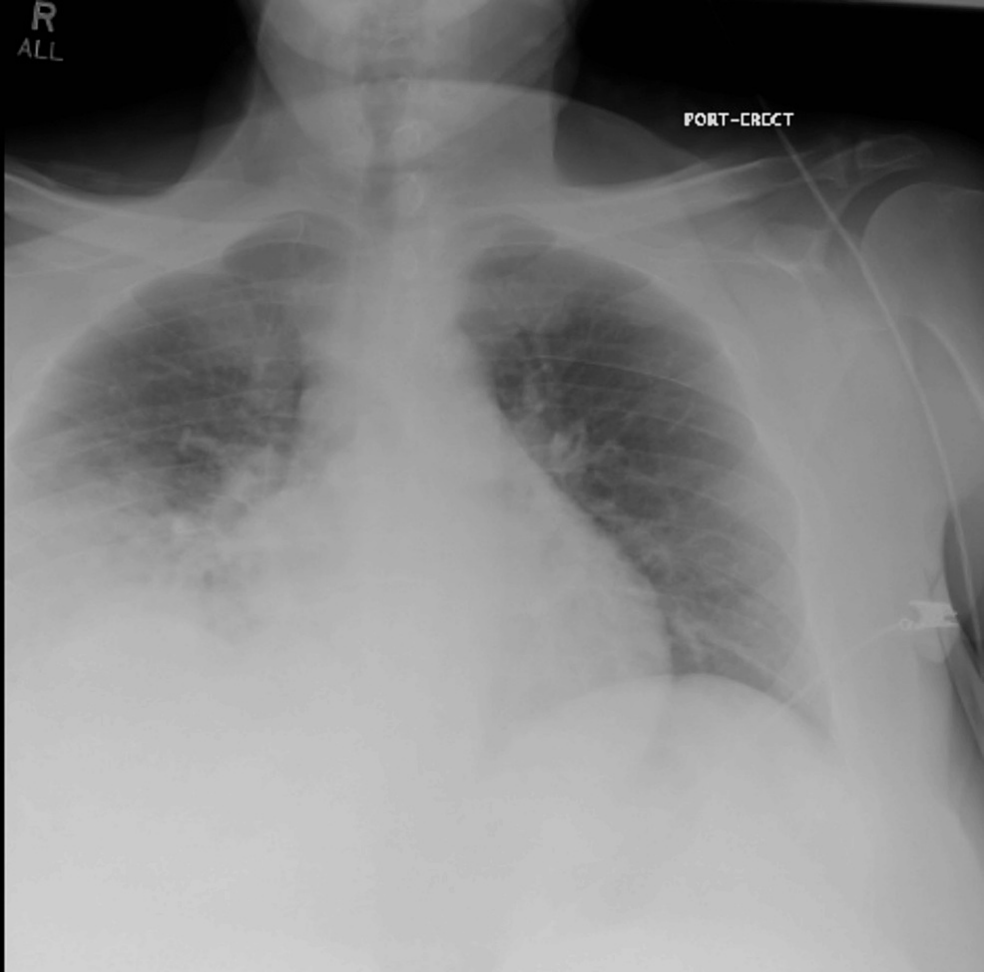
Chest X-ray obtained at the ER on day 1 shows right lower lobe infiltrates and small right-sided pleural effusion.

**Figure 2:**
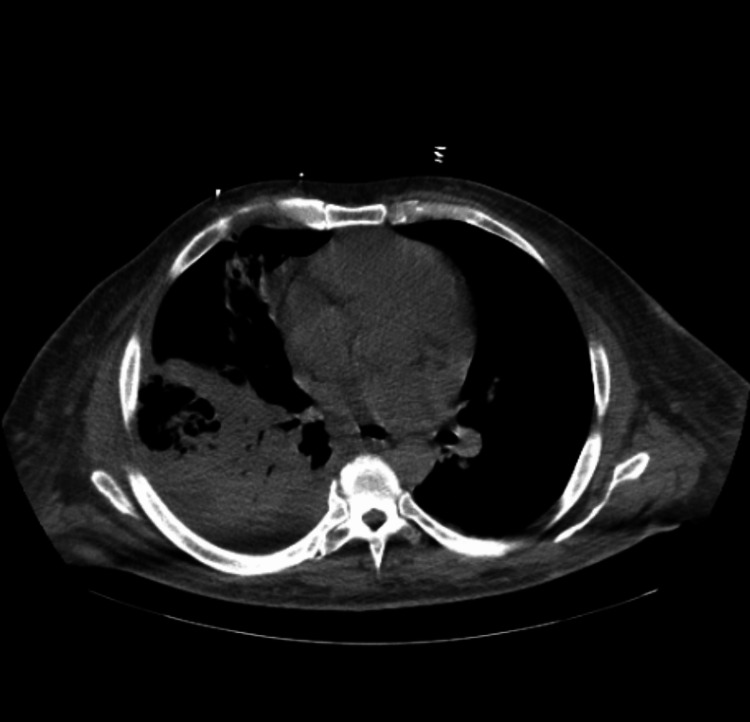
Chest/abdomen/pelvis CT without contrast taken on day 1 reveals right loculated pleural effusion and lung abscess.

His CT head without contrast showed findings of sulcal effacement and partially effaced basal cisterns which was suspicious for cerebral edema. Chest X-ray and chest/abdomen/pelvis CT revealed dense right middle lobe and right lower lobe consolidation with air bronchograms, suspicious for lobar pneumonia, small right pleural effusion (Figure 1 and Figure 2), moderate anasarca, and cystitis.

The patient's labs included a complete blood count and comprehensive metabolic panel as shown in Table 1 and Table 2.

**Table 1:** The patient's complete blood count.

Complete blood count	Reference ranges	Patient's lab values
White blood cell count	4.5-12.5x10^3^/uL	8.5x10^3^/uL
Red blood cell count	4.70-6.10x10^6^/uL	4.79x10^6^/uL
Hemoglobin	13.5-18.0 g/dL	15.0 g/dL
Hematocrit	40.5-54.0%	41.9%
Mean corpuscular volume	80.0-95.0 fL	87.5 fL
Mean corpuscular hemoglobin concentration	31.0-36.0 g/dL	35.8 g/dL
Platelets	150-450x10^3^/uL	107x10^3^/uL

**Table 2:** The patient's comprehensive metabolic panel.

Comprehensive metabolic panel	Reference ranges	Patient's lab values
Sodium	136-145 mmol/L	126 mmol/L
Potassium	3.4-4.9 mmol/L	2.9 mmol/L
Chloride	98-107 mmol/L	96 mmol/L
Carbon dioxide	21-32 mmol/L	15 mmol/L
Anion gap	1-11 mmol/L	15 mmol/L
Blood urea nitrogen	7-25 mg/dL	47 mg/dL
Creatinine	0.60-1.30 mg/dL	3.40 mg/dL
Estimated glomerular filtration rate	>60.0 mL/min/1.73m^2^	22.1 mL/min/1.73m^2^
Glucose, random	74-109 mg/dL	70 mg/dL
Calcium	8.6-10.2 mg/dL	7.2 mg/dL
Alkaline phosphatase	30-105 U/L	43 U/L
Albumin	3.5-5.7 g/dL	3.1 g/dL
Total protein	6.4-8.9 g/dL	5.1 g/dL
Aspartate aminotransferase	13-39 U/L	262 U/L
Alanine transaminase	7-52 U/L	116 U/L
Bilirubin direct	0.03-0.18 mg/dL	0.82 mg/dL
Bilirubin indirect	0.2-0.8 mg/dL	2.28 mg/dL
Bilirubin total	0.3-0.1 mg/dL	3.1 mg/dL
Lactate	0.5-2.0mmol/L	6.2 mmol/L
Magnesium	1.9-2.7 mg/dL	1.2 mg/dL
Glucose	74-106 mg/dL	70 mg/dL

 Other labs including thyroid panel and iron panel are shown in Table 3.

**Table 3:** Other labs, thyroid panel, and iron panel.

Other labs	Reference ranges	Patient's lab values
Serum osmolality	280-295 mOsm/kg	275 mOsm/kg
Urine osmolality	500-800 mOsm/kg	344 mOsm/kg
Urine sodium	20-30 mmol/L	31 mmol/L
Lipase	11-82 U/L	12 U/L
Lactate dehydrogenase	135-275 U/L	857 U/L
Vitamin B1	66.5-200 nmol/L	231.1 nmol/L
Vitamin B12	180-914 pg/mL	5,278 pg/mL
Copper	69-132 ug/dL	82 ug/dL
Zinc	44-115 ug/dL	30 ug/dL
Iron panel	Reference ranges	Patient's lab values
Unsaturated iron-binding capacity	155-355 ug/dL	120 ug/dL
Iron	50-212 ug/dL	19 ug/dL
Iron saturation	15-50%	14%
Ferritin	23.9-336.2 ng/mL	1003.4 ng/mL
Thyroid studies	Reference ranges	Patient's lab values
Thyroid-stimulating hormone	0.450-5.330 ulU/mL	1.532 ulU/mL
Free T4	0.61-1.12 ng/dL	0.90 ng/dL

Urine was dark and minimal, and results of the patient's urinalysis are shown in Table 4.

**Table 4:** Results of the patient's urinalysis. RBC: red blood cell; WBC: white blood cell

Urinalysis	Reference ranges	Patient's lab values
Color, urine	4.5-12.5x10^3^/uL	Yellow
Clarity, urine	4.70-6.10x10^6^/uL	Densely turbid
Specific gravity, urine	1.010-1.025	1.018
pH, urine	5.0-8.0	5.0
Leukocytes, urine	Negative	Trace abnormal
Nitrite, urine	Negative	Negative
Protein, urine	Negative	1+ abnormal
Glucose, urine	Negative	Negative
Ketones, urine	Negative	Trace abnormal
Urobilinogen, urine	Normal	Normal
Bilirubin, urine	Negative	Negative
Blood, urine	Negative	3+ abnormal
RBC, urine	0-3 HPF	3 HPF
WBC, urine	0-10 HPF	9 HPF
Squamous epithelial, urine	<15-20 HPF	2 HPF
Bacteria, urine	None seen	Occasional
Trans-epithelial, urine	0 HPF	<1 HPF
Mucus, urine	None seen	1+
Granular casts, urine	<1 HPF	11 HPF
Hyaline casts, urine	<1 LPF	11 LPF

His electrocardiogram revealed sinus tachycardia with a rate of 137 beats per minute, with no ST elevation or depression. His quick sequential organ failure assessment (qSOFA) score was greater than 2 so he was immediately started on vancomycin, cefepime, Flagyl, and acyclovir. His magnesium and potassium were also repleted in the ER. He received 6 liters of crystalloids, and he was started on epinephrine once he maxed out on the initial two vasopressors (Levophed and vasopressin). Despite these interventions, he remained hypotensive with an IVC <1.8 cm with >50% collapse on point-on-care ultrasound. He was admitted to the intensive care unit (ICU) for the further management of suspected septic shock and cerebral edema of unknown etiology.

**Figure 3:**
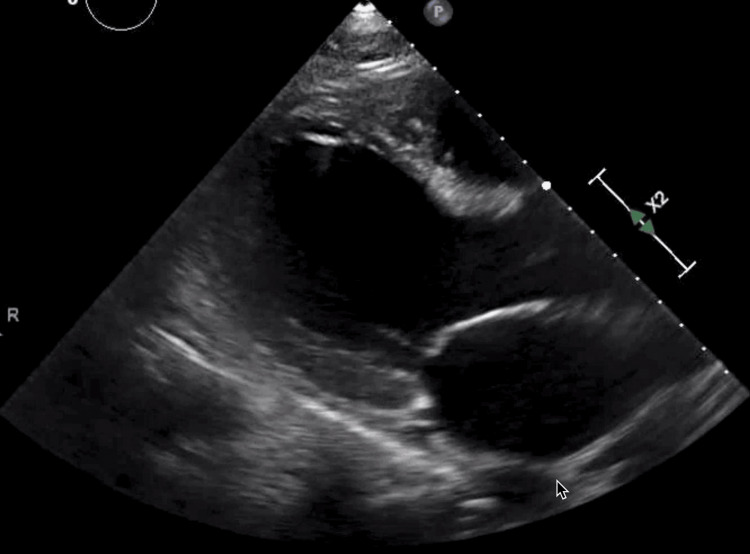
Transthoracic echocardiogram taken on day 1 post-admission shows a severely enlarged left ventricle with normal wall thickness and moderate global hypokinesis and mildly enlarged left atrium with an ejection fraction of 35-40%.

His urine drug screening was positive for benzodiazepine and opiate which he received in the ER. His creatinine kinase was 8,401 U/L and initial troponin was 764 pg/mL which plateaued at 16,804 pg/mL within 24 hours and then trended down to 12,619 pg/mL. His transthoracic echocardiography (TTE) showed cardiomyopathy of suspected septic origin with an ejection fraction of 35-40% with mildly enlarged atrium, severe left ventricular enlargement with normal wall thickness, and moderate global hypokinesis (Figure 3).

His blood culture, SARS-CoV-2/flu/RSV, MSRA PCR, HIV, HAV, HBV, HCV, *Streptococcus pneumoniae*, and *Chlamydia pneumoniae* were negative, and his respiratory culture showed no growth on day 3. However, his nasopharyngeal swab sample for respiratory pathogen panel via polymerase chain reaction detected hMPV.

**Figure 4:**
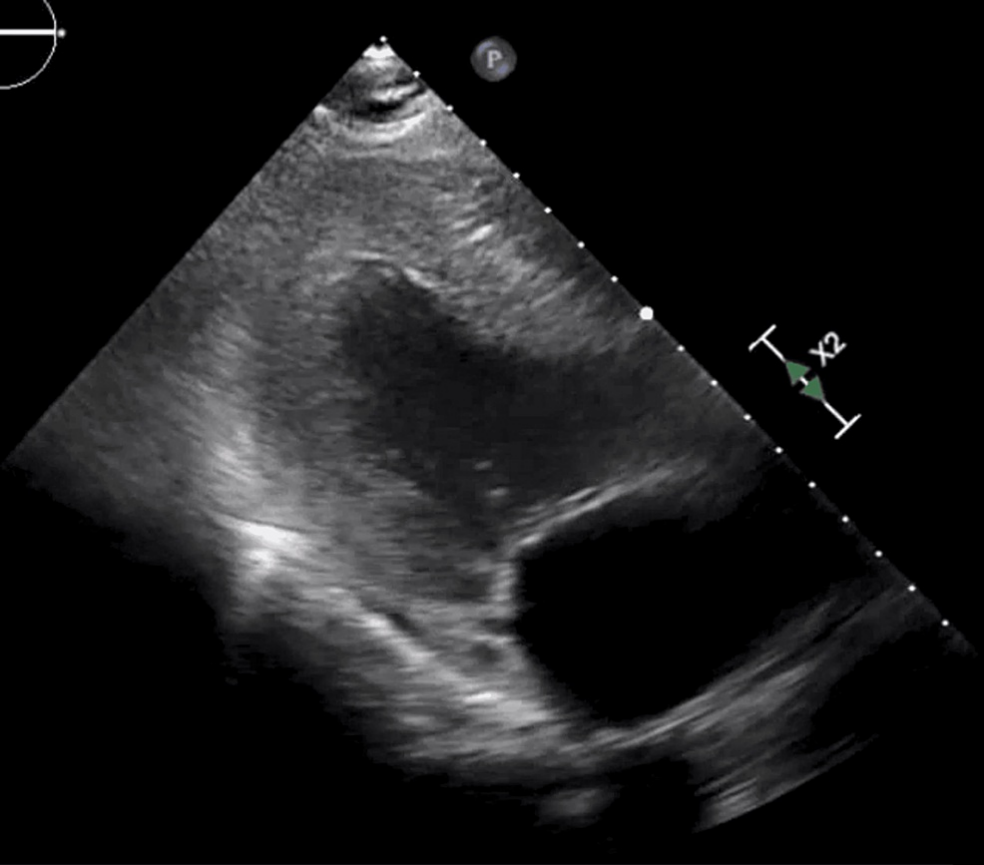
Transthoracic echocardiogram taken in the ICU on day 9 post-admission shows a normal left ventricle with an improved ejection fraction of 50-55%.

He was diagnosed with SICM secondary to hMPV and managed in the ICU for sepsis. His repeated TTE done on day 9 post-admission showed an improved ejection fraction close to his baseline of 50-55% (Figure 4).

**Figure 5:**
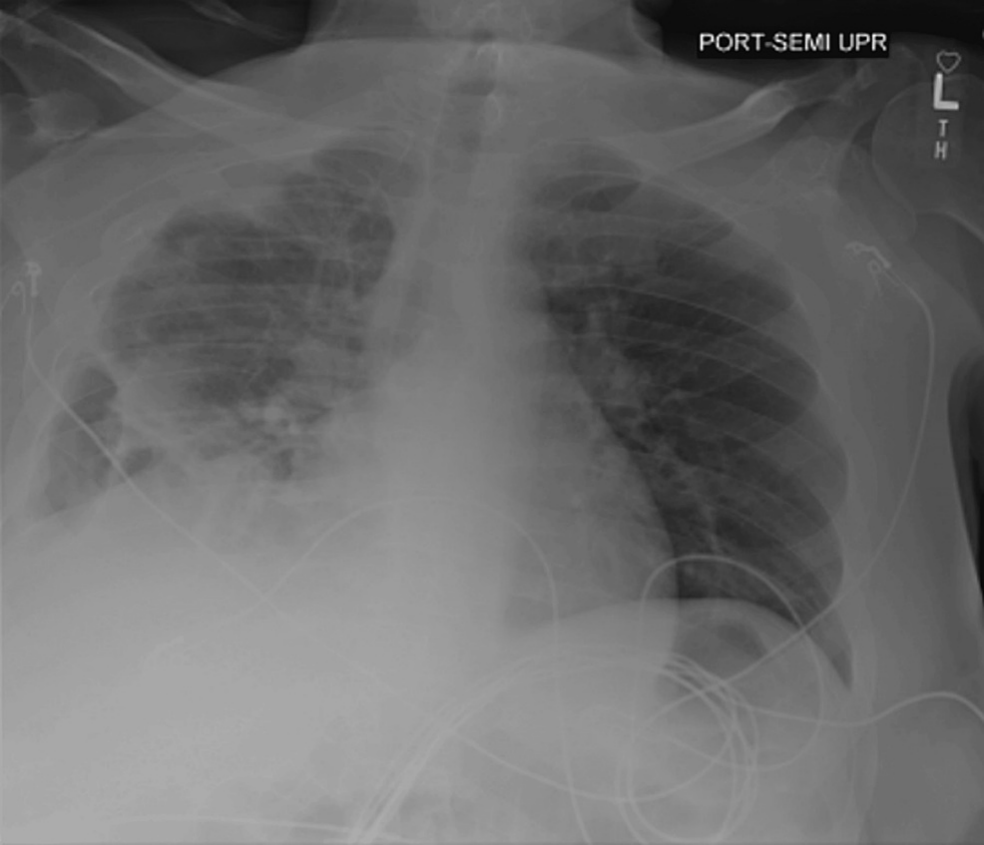
Chest X-ray taken on day 20 shows persistent pleural-based opacity in the right lung apex and persistent consolidation at the right lung base after chest tube removal.

Given his improved cardiac function, we did not initiate any guideline-directed medical therapy (GDMT) during his hospitalization. Subsequent chest X-ray and chest CT with contrast done on day 10 revealed right-sided loculated pleural effusion and lung abscess. He underwent right-sided thoracotomy and decortication with chest tube placement which was subsequently removed on day 20 (10 days post-chest tube insertion). The subsequent chest X-ray done on day 20 showed improved lung functions (Figure 5). Fluid cultures and blood cultures from the lung samples showed no growth to date. He was prescribed Zosyn for six weeks from the date of decortication through a peripherally inserted central catheter. He was admitted into the inpatient acute rehabilitation on day 24 post-admission and completed 16 days of rehab before he was discharged home in stable condition on day 40 post-admission. 

## Discussion

SICM is a complication of sepsis and septic shock characterized by left ventricular dilation and depressed ejection fraction, with recovery in 7-10 days without treatment [1]. Chemical mediators have been proposed as one of the possible causative mechanisms causing cardiac dysfunction [1]. In SICM, the myocardium is functionally and structurally injured by inflammatory cytokines and mitochondrial dysfunction [1]. This results in decreased ejection fraction as a result of decreased myofibril response to calcium as well as downregulation of beta-adrenergic receptors.

hMPV is a known major cause of self-limiting upper and lower respiratory infections in both children and adults [2]. It is spread from person to person via respiratory droplets and has an incubation period of 3-5 days although this varies between individuals [10]. Its pathogenesis results in the production of several chemical mediators [2]. These chemokines include interleukin-6, interferon-alpha, tumor necrosis factor-alpha, and interleukin-2, as well as macrophage inflammatory proteins leading to peribronchiolar and perivascular infiltration and inflammation [10]*.* Extra-pulmonary manifestations of hMPV, including cardiac complications, have been reported in other case reports [4,6-8]. Cardiovascular complications have been seen as more common in hMPV infections as compared to other respiratory viruses like influenza infection [11]. Whether hMPV has an affinity for myocardium or if patients with prior underlying cardiovascular disease are more susceptible to hMPV remains to be clarified [4].

Our patient presented with septic shock with acute onset of dilated cardiomyopathy after two days of upper respiratory tract infection symptoms. He was subsequently found to have right lung loculated pleural effusion and abscess. His TTE showed cardiomyopathy with an ejection fraction of 35-40% and mildly enlarged atrium, severe left ventricular enlargement with normal wall thickness, and moderate global hypokinesis which is consistent with sepsis-induced dilated cardiomyopathy. His nasopharyngeal sample tested positive for hMPV, while his blood and respiratory culture remained negative during his hospital stay. Although his stay was complicated by pleural effusion and pulmonary abscess requiring a chest tube and thoracotomy with decortication as a complication of his respiratory infection, his cardiac function recovered within eight days, and his ejection fraction improved from 35-40 % to 50-55 % without the use of GDMT.

The rapid recovery of our patient's ejection fraction indicates his depressed cardiac function was directly related to sepsis secondary to infection with hMPV. 

## Conclusions

The case is a classic presentation of SICM that resolved within 7-10 days with the return to baseline ejection fraction of 50-55%. The detection of hMPV could help explain the etiology of the chemical mediators that contributed to dilated cardiomyopathy. The association between hMPV and dilated cardiomyopathy and its subsequent implications and clinical relevance warrants further research.
